# Nonlinear saturable absorption properties of BP/ReS_2_ heterojunction and its application in 2 μm all-solid-state lasers

**DOI:** 10.1007/s12200-025-00157-3

**Published:** 2025-06-11

**Authors:** Hongqing Li, Wenjing Tang, Yingshuang Shan, Jing Wang, Kai Jiang, Mingqi Fan, Tao Chen, Cheng Zhou, Wei Xia

**Affiliations:** 1 School of Physics and Technology, University of Jinan, Jinan, 250022 China; 2 Shandong Huaguang Optoelectronic Co., Ltd, Jinan, 250013 China; 3 School of Information Science and Engineering, Shandong University, Jinan, 250100 China; 4 Wuhan Huaray Precision Laser Co., Ltd, Wuhan, 430000 China

**Keywords:** BP/ReS_2_, Heterojunction, Saturable absorption, All-solid-state laser, Q-switched

## Abstract

For 2 μm all-solid-state lasers, pulse modulation methods based on low-dimensional nanomaterial saturable absorbers (SAs) offer advantages such as compact structure, low cost, and ease of implementation. The construction of stable, highly nonlinear low-dimensional nanomaterial SAs is an urgent issue to be addressed. In this paper, two types of black phosphorus/rhenium disulfide (BP/ReS_2_) heterojunction with high stability were prepared separately by liquid phase exfoliation (LPE) and mechanical exfoliation (ME) methods, the nonlinear saturable absorption characteristics of the two types of heterojunctions have been characterized in detail. Then, the pulse modulation applications of these two materials have been studied in a 2 μm all-solid-state thulium-doped yttrium aluminum perovskite (Tm:YAP) passively Q-switched pulsed laser. The BP/ReS_2_ heterojunction SA prepared by the LPE method demonstrates a thinner thickness and lower non-saturation optical loss, which achieved the maximum average output power 528 mW at a pump power of 6.37 W, with a narrowest pulse width of 366 ns, and a maximum peak power of 28.85 W. These results indicate that the BP/ReS_2_ heterojunction SA has great potential for optical modulation device applications.

**Graphical Abstract:**

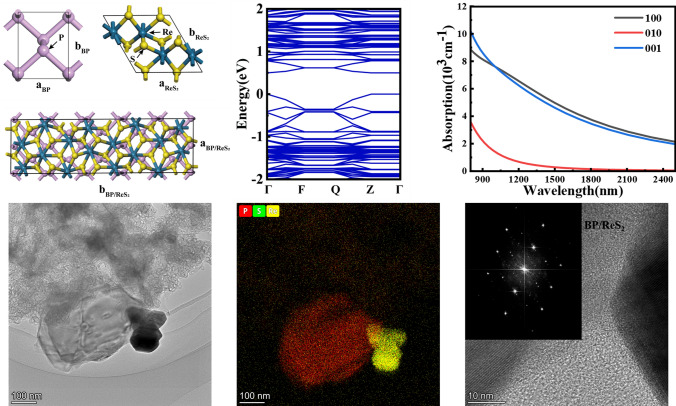

## Introduction

The 2 μm all-solid-state pulse lasers with high peak power are highly favored due to their unique characteristics, such as high beam quality, strong absorption of gas molecules, and operation within the eye-safe wavelength range. These distinctive attributes ensure their secure utilization in various domains, including material processing, light detection and ranging technology, medical surgeries, and numerous other applications [1–3]. Currently, the passive Q-switching technology based on saturable absorbers (SAs) is the most mature and commonly utilized technique for achieving 2 μm high peak power pulses in all-solid-state lasers for its features of efficiency, compactness, and cost-effectiveness [4]. In this category of laser systems, the SA material plays an invaluable role in optimizing the quality of laser pulses. However, there is a limited variety of traditional SA materials suitable for the 2 μm wavelength band. Therefore, the exploration of high-quality SA materials has become one of the research hotspots in the laser field.

Nowadays, two-dimensional (2D) materials used as SAs have been extensively studied and developed rapidly due to their broadband absorption, large specific surface area, and superior saturable absorption properties (such as ultra-fast recovery time, controllable modulation depth, and low non-saturation loss) [5–9]. Commonly used 2D materials include black phosphorus (BP), graphene, carbon nanotubes (CNTs), topological insulators (TIs), and transition metal dichalcogenides (TMDCs) [10–15]. All of them have been successfully applied in all-solid-state or fiber lasers for pulse modulation [16–22]. Very recently, topological insulator Bi_2_Se_3_ and transition metal trichalcogenide HfS_3_ materials demonstrating exceptional nonlinear optical properties and high stability have been successively reported. Based on these two materials, ultrashort pulse with durations of 195 and 540 fs were achieved in erbium-doped fiber lasers, respectively, highlighting the considerable potential of 2D materials as advanced pulse modulators in photonic applications [21, 22]. However, in all-solid-state pulsed lasers, the application of 2D material-based SAs still faces significant challenges. Because of the large pulse energy, the damage thresholds, stability, and service life of single 2D material films need to be optimized. Therefore, exploring SA materials with better stability and superior nonlinear saturable absorption properties is an urgent issue in the field of all-solid-state lasers.

The research of 2D heterojunction materials provide a new solution to the problem [23–25]. According to previous reports, 2D heterostructures materials not only retain and optimize the broadband and nonlinear optical properties of single 2D material, but also significantly enhance the material's stability and damage threshold [26–28]. However, as of now, the exploration of 2D heterostructure materials suitable for mid-infrared all-solid-state lasers is still in its early stages. The construction of heterostructure materials with excellent nonlinear saturable absorption mechanism for 2 μm pulse modulation remains to be further explored.

Among numerous 2D materials, BP and TMDCs have exhibited excellent saturable absorption properties and have been successfully applied in 2 μm pulsed lasers [29–31]. BP exhibits a broadly tunable direct bandgap ranging from 0.3 eV (bulk structure) to 2.0 eV (monolayer BP) coupled with high carrier mobility and switching ratios, which has been extensively employed in mid-infrared pulsed laser applications. However, how to enhance the stability of BP nanosheets is an urgent problem. Rhenium disulfide (ReS_2_), as a member of TMDCs family, exhibits a distorted 1 T phase and demonstrates unique direct bandgap characteristics, maintaining exceptional physicochemical stability in both 2D and three-dimensional (3D) configurations, which makes its stability significantly superior to that of other TMDCs materials [32, 33]. Compared to other TMDCs materials, the interlayer coupling in ReS_2_ via van der Waals forces is weaker, which makes it advantageous for the formation of high-quality heterojunctions. Furthermore, the large bandgap of both BP and ReS_2_ are conducive to achieving a higher optical transmittance, thereby enhancing the material’s resistance to damage [21]. In 2018, black phosphorus/rhenium disulfide (BP/ReS_2_) heterojunctions was constructed and studied by Cao et al. Their research results indicated that BP/ReS_2_ heterojunctions possessed excellent interfacial quality without lattice mismatch issues, exhibiting outstanding electronic and optoelectronic properties [34]. Subsequently, research on the optical response and optoelectronic properties of BP/ReS_2_ heterojunctions was also conducted through both theoretical and experimental approaches [35–37]. Those reports demonstrate that BP/ReS_2_ heterojunctions have great research value as SAs for pulses modulation. However, up to now, the nonlinear saturable absorption characteristics of BP/ReS_2_ heterojunctions and its application in mid-infrared pulse modulation have yet to be reported.

In this paper, based on the first principles, the energy band structure and optical properties of BP/ReS_2_ heterojunction were calculated and analyzed. Then, using liquid phase exfoliation (LPE) and mechanical exfoliation (ME) methods, two types of BP/ReS_2_ heterojunction SAs were prepared respectively. Based on these two types of SAs, a 2 μm all-solid-state thulium-doped yttrium aluminum perovskite (Tm:YAP) passively Q-switched pulsed laser was constructed. The nonlinear saturable absorption properties of BP/ReS_2_ heterojunction have been systematically studied and presented. The output power, pulse width, repetition frequency, and other parameters of the pulsed laser were also investigated meticulously.

## Preparation and characterization of BP/ReS_2_ heterojunction

### Preparation

**Fig. 1:**
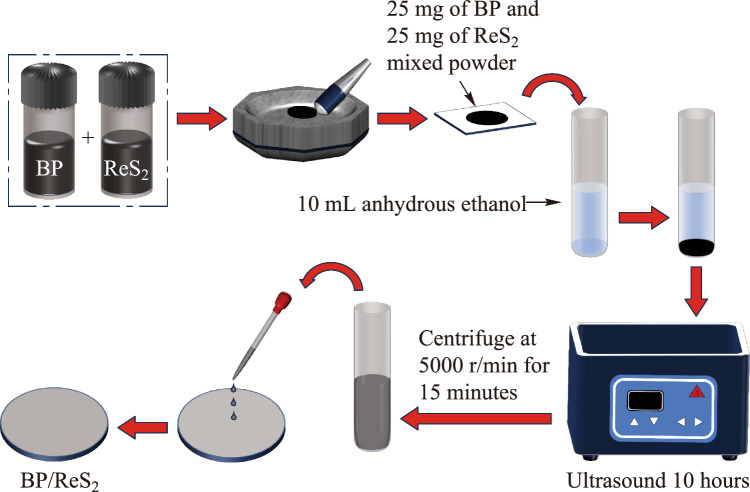
Flow chart of BP/ReS_2_ heterojunction preparation by LPE method

To obtain better pulse modulation effects, we prepared BP/ReS_2_ heterojunction SAs using both the LPE method and the ME method, respectively. And two types of BP/ReS_2_ heterojunction SAs were correspondingly designated as SA-LPE and SA-ME in this paper. The specific procedure for preparing SA-LPE is shown in Fig. 1. First, 25 mg BP power was mixed with 25 mg ReS_2_ powder and thoroughly ground in a mortar and pestle. Second, the ground powder was mixed with 10 mL alcohol and ultrasounded in a sonicator for 10 h. Then, the suspension was centrifuged at 5000 r/min for 15 min to sieve out bulky particles, resulting in a BP/ReS_2_ nanosheet dispersion. Finally, the supernatant was taken and dropped onto a clean sapphire substrate to produce a BP/ReS_2_ SA.

**Fig. 2:**
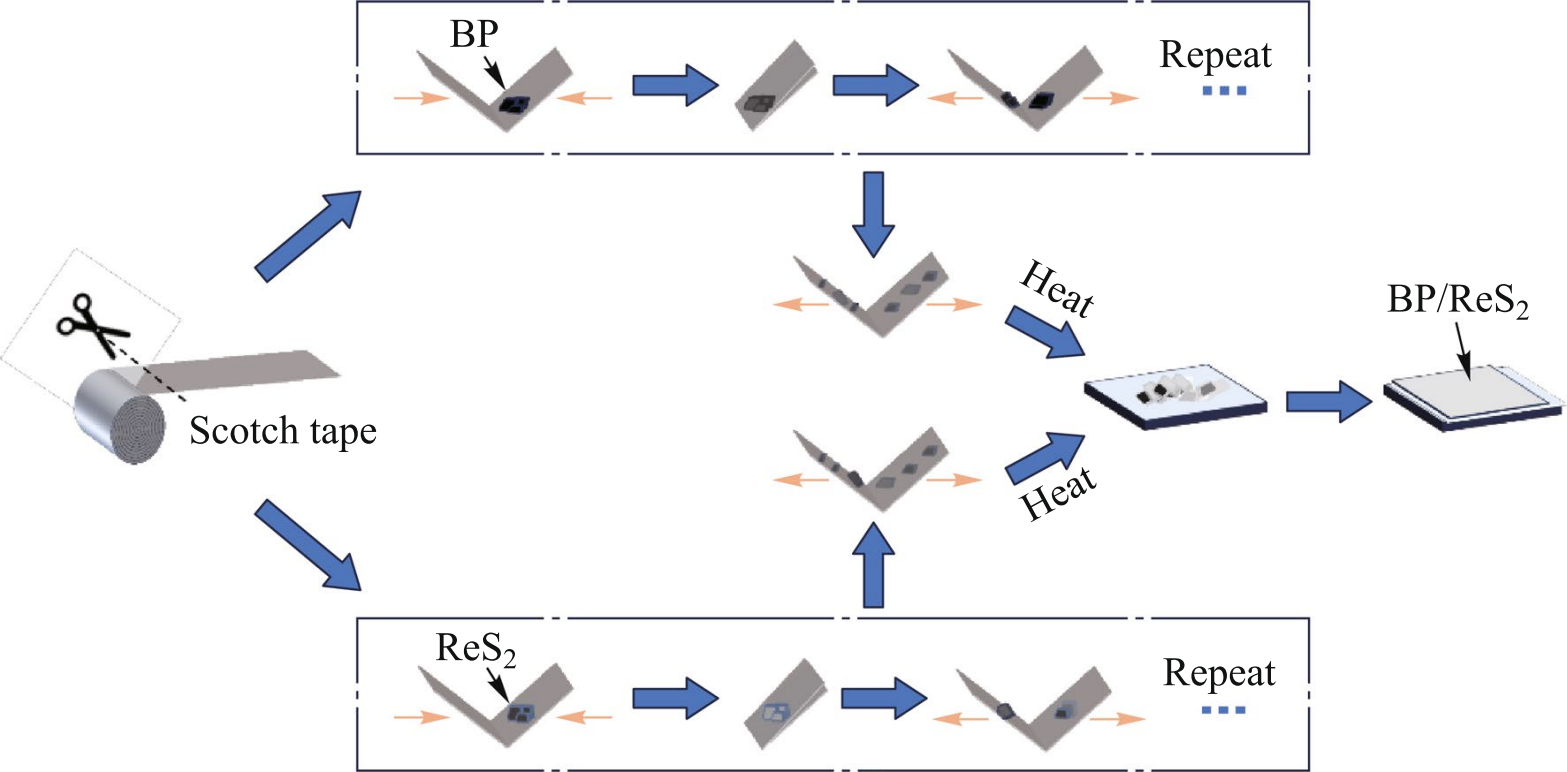
Flow chart of BP/ReS_2_ heterojunction preparation by ME method

The preparation procedure of SA-ME is illustrated in Fig. 2. First, high-quality BP or ReS_2_ crystals were placed on Scotch tape. Next, pressure was applied to make the tape fit tightly on the surface of the material, and then the tape was gently peeled off. This process was repeated several times until a sheet with a thickness of about 3 nm was obtained. The BP or ReS_2_ lamellar flakes were then transferred onto an SiO_2_ substrate using heat. Finally, under the observation of an optical microscope, the position of the BP or ReS_2_ lamellae was precisely controlled using microtubules so that the contact area of the two was as large as possible. They were attracted to each other through van der Waals forces to form BP/ReS_2_ heterojunctions.

### Characterization

#### Theoretical calculation

Theoretical calculation of the energy band structure and optical properties of BP/ReS_2_ were performed based on density functional theory using the CASTEP module in Material Studio software. The exchange–correlation potential between interacting electrons was described by the Perdew–Burke–Ernzerhof (PBE) generalization of the generalized gradient approximation (GGA).

**Fig. 3:**
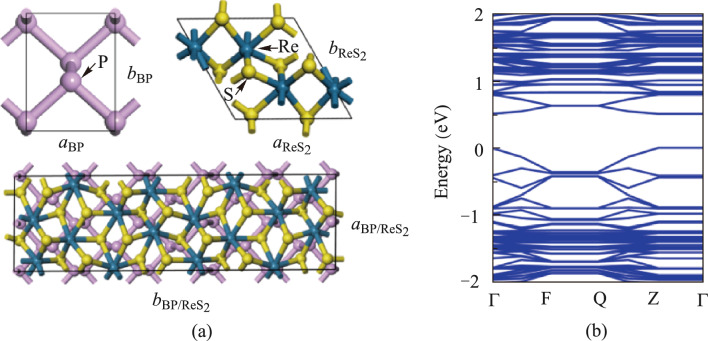
**a** Atomic structure diagrams of BP, ReS_2_ and BP/ReS_2_ heterojunction. **b** Energy band structure diagrams of BP/ReS_2_ heterojunction

ReS_2_ exhibits weaker van der Waals interlayer forces and a larger layer spacing compared to other common TMDCs [38]. This weaker interlayer force can help reduce interfacial defects and stresses which may occur in BP/ReS_2_ heterojunctions. This facilitates the formation of stable and high-quality heterostructures. The structures of BP and ReS_2_ are shown in Fig. 3a, respectively. After lattice optimization, the lattice constants of monolayer BP are *a* = 3.31 Å and *b* = 4.38 Å. The lattice constants of monolayer ReS_2_ are *a* = 6.56 Å and *b* = 11.11 Å. To satisfy the lattice match rate, a 2 × 5 × 1 BP unit cell was used to match a 1 × 2 × 1 ReS_2_ unit cell to construct the heterojunction. Geometric optimization of the BP/ReS_2_ heterojunction was performed using 5 × 1 × 1 *k*-point sampling, with a cutoff energy of 500 eV. Through structural optimization, the lattice constants of the BP/ReS_2_ heterojunction supercell were determined to be *a* = 6.6 Å and *b* = 22.35 Å, as shown in Fig. 3a. The optimal interlayer spacing calculated after lattice relaxation is 4.13 Å.

According to previous reports, the bandgap of ReS_2_ is approximately 1.6 eV, while the bandgap of BP is tunable from 0.3 to 2.0 eV [39, 40]. The band structure of the BP/ReS_2_ heterojunction is calculated using the GGA-PBE method and depicted in Fig. 3b. The bandgap of BP/ReS_2_ heterojunction we constructed is 0.498 eV. The calculated energy-band bandgap value for BP/ReS_2_ heterojunction is different from individual materials (monolayer BP and monolayer ReS_2_), indicating that the electronic energy bands of these two components undergo restructuring at the interface and forming a new band structure. This band restructuring might lead to localized electron states and charge transfer at the interface, potentially impacting both the electronic and optoelectronic properties of the BP/ReS_2_ heterojunction significantly [41, 42]. This indicates that the 2D BP can compensate for the gap of ReS_2_, enabling the formation of BP/ReS_2_ heterojunctions with various bandgap values. Such heterojunctions have potential applications in broadband pulse modulation, as they can customize the bandgap of the heterojunction by adjusting the bandgap of BP.

**Fig. 4:**
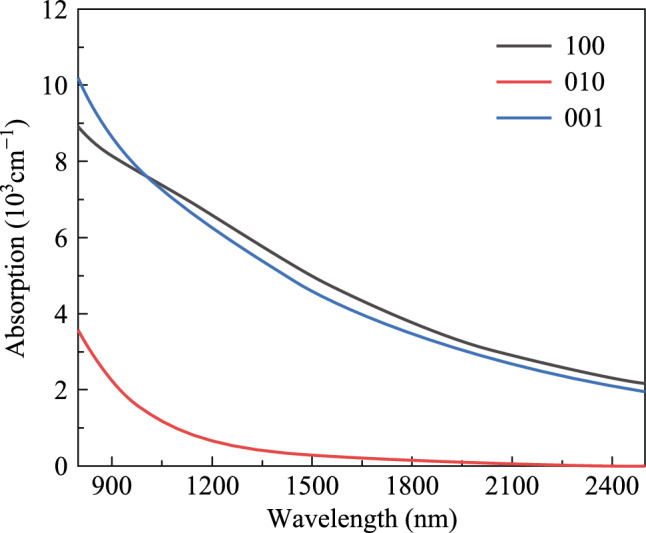
Absorption spectra of BP/ReS_2_ heterojunction along three directions

To further elucidate the optical absorption properties of the BP/ReS_2_ heterojunction, the absorption spectra along the [1 0 0], [0 1 0], and [0 0 1] directions of the BP/ReS_2_ heterojunction were calculated, as shown in Fig. 4. The absorption values at 2 μm along the [1 0 0], [0 1 0], and [0 0 1] directions are 3140, 81.5, and 2893 cm^−1^, respectively. It is evident that the absorption in the [010] direction of the BP/ReS_2_ heterojunction differs significantly from the other two directions, indicating high anisotropy. The absorption window in the wavelength range of 800–2500 nm only permits light polarization in the *X* and *Z* directions, with transitions in the *Y* direction being prohibited. When employed as a SA in 2 μm all-solid-state lasers, the BP/ReS_2_ heterojunction not only generates short, high-energy pulses but also ensures that the laser radiation possesses the desired polarization state through its polarization selectivity. This is particularly crucial in fields such as military applications and material processing.

#### Structural and morphological characterization

**Fig. 5:**
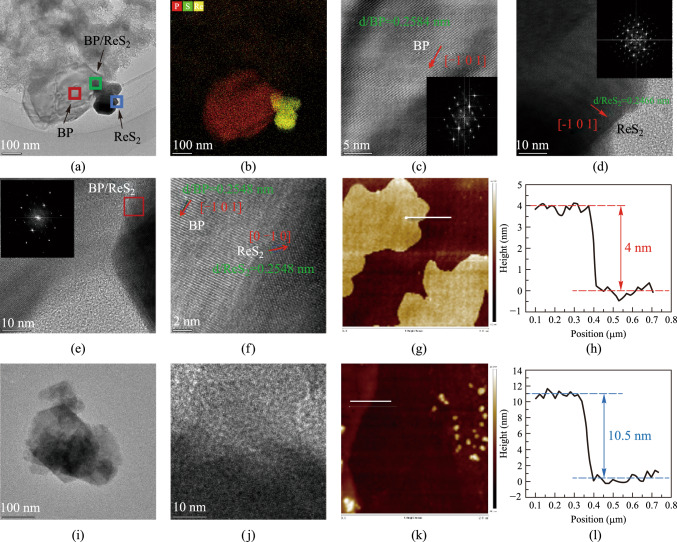
**a** TEM image (100 nm). **b** EDS elemental mapping. **c** HRTEM image of BP nanosheets (5 nm). **d** HRTEM image of ReS_2_ nanosheets (10 nm). **e** HRTEM image of BP/ReS_2_ heterojunction (10 nm). **f** HRTEM image of BP/ReS_2_ heterojunction (2 nm). **g**, **h** AFM image and the corresponding thickness distribution curve of SA-LPE nanosheets. **i** TEM image (100 nm). **j** HRTEM image of BP/ReS_2_ heterojunction (10 nm). **k**, **l** AFM image and the corresponding thickness distribution curve of SA-ME nanosheets

For the two preparation methods, we conducted systematic morphological and structural characterization of the samples. Figure 5a–h show the characterization results of SA-LPE, while Fig. 5i−l present the results of SA-ME. Figure 5a presents the transmission electron microscopy (TEM) image of the SA-LPE sample, while Fig. 5b shows the corresponding energy dispersive spectroscopy (EDS) elemental mapping. The EDS analysis confirms the coexistence of BP and ReS_2_ nanosheets in the sample. To conduct an in-depth analysis of the material’s morphological and structural characteristics, high-resolution transmission electron microscopy (HRTEM) tests were performed on the regions corresponding to the red, green, and blue squares marked in Fig. 5a. Corresponding FFT processing was also applied to the HRTEM images. The measurement results are respectively shown in Fig. 5c–e. The single BP nanosheet exhibits an interplanar spacing of 0.2584 nm with a crystal orientation of [− 1 0 1], while the single ReS_2_ nanosheet demonstrates an interplanar spacing of 0.2466 nm with an identical crystal orientation of [− 1 0 1]. Figure 5e and f present the HRTEM images with different resolutions of the BP/ReS_2_ heterojunction region. The HRTEM image with 2 nm resolution was recorded according to the red box area in Fig. 5e and shown in Fig. 5f. It is worth noting that when the two materials overlap to form a heterojunction, the interplanar spacing of both BP and ReS_2_ become the same 0.2548 nm, indicating that both materials undergo lattice distortion. Moreover, the crystal orientation of the ReS_2_ nanosheets in the heterojunction region is [0 − 1 0], which is different from that in the region far away from the heterojunction. This is mainly due to the lattice mismatch between the ReS_2_ nanosheets and the BP nanosheets. The TEM image and HRTEM images of SA-ME were also conducted and shown in Fig. 5i and j, respectively. The BP/ReS_2_ heterojunction nanosheets’ topography of the SA-LPE and the SA-ME were also be obtained by atomic force microscopy (AFM) measurements, as shown in Fig. 5g and k. The corresponding thickness distribution curves of the samples are shown in Fig. 5h and l, respectively. It can be seen that the average thickness of the samples at the white line is 4 and 10.5 nm, respectively.

**Fig. 6:**
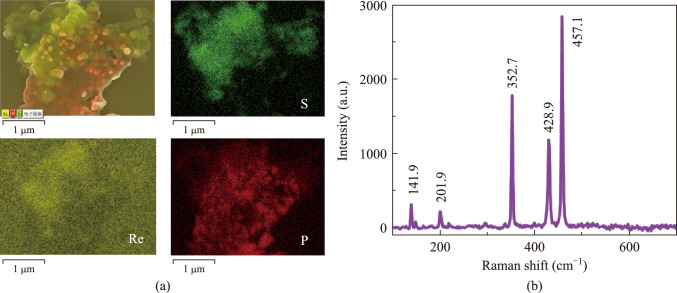
**a** EDS images (elemental species) of SA-LPE nanosheets. **b** Raman spectrum of SA-LPE nanosheets

Here, we find that the thickness of the nanosheets prepared by the LPE method is significantly smaller than that by the ME method, and the formation of the heterojunction cannot be precisely controlled. To identify the composition of the nanosheets obtained by the LPE method, Raman spectroscopy and EDS testing were also carried out, with the test results presented in Fig. 6. Figure 6a shows the EDS analysis of the elemental species in the microzone of the sample. The distribution of Re, P, and S in the BP/ReS_2_ heterojunction sample can be observed, indicating that the BP and ReS_2_ nanosheets are well combined to form a heterojunction. The Raman spectra was shown in Fig. 6b. By comparing the Raman spectra of BP and BP/ReS_2_ heterojunctions [43], it can be observed that the peaks at 352.7, 428.9, and 457.1 cm^−1^ corresponding to the A_1__g_, B_2g_, and A_2__g_ modes of BP which has been reported many times, respectively, and the peaks are not shifted. Similarly, by comparing Fig. 6b with the Raman spectra of ReS_2_ [44], it was found that the peak positions of BP/ReS_2_ and ReS_2_ were the same at 141.9 and 201.9 cm^−1^, respectively. This indicates that BP and ReS_2_ are stacked in the vertical direction to form a BP/ReS_2_ heterojunction and maintain a good structural match.

#### Nonlinear saturable absorption characteristics

**Fig. 7:**
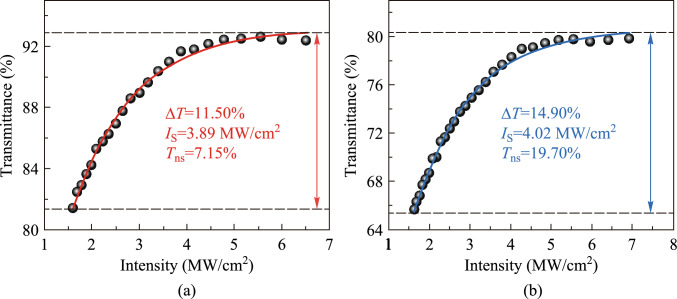
Nonlinear transmittance curves of **a** SA-LPE and **b** SA-ME

To further investigate the nonlinear absorption effect of the BP/ReS_2_ heterojunction, *Z*-scan tests for nonlinear transmittance were conducted separately for the two SAs. The self-constructed 1 μm all-solid-state pulsed laser is used as the light source for material testing. By controlling the position of the sample on the *Z*-axis, the nonlinear transmittance *T*(*I*) of the BP/ReS_2_ heterojunction can be measured. The nonlinear transmittance curve of SA-LPE and SA-ME are shown in Fig. 7a and b, respectively, which were fitted by the following equation.

$$T\left(I\right)=1-{T}_{\text{ns}}-\Delta T\text{exp}\left(-\frac{I}{{I}_{\text{S}}}\right),$$

where the input light intensity of the laser is *I*, and the modulation depths ∆*T* of the two BP/ReS_2_ heterojunctions SAs are 11.50% and 14.90%, respectively. The saturation light intensities *I*_S_ of SA-LPE and SA-ME are 3.89 and 4.02 MW/cm^2^, corresponding to the non-saturated losses *T*_ns_ of 7.15% and 19.70%, respectively. The BP/ReS_2_ heterojunction SA prepared by the LPE method demonstrates a thinner thickness and lower non-saturation optical loss, while the SA-ME has larger modulation depth and non-saturation optical loss. This is mainly caused by the different thicknesses of the two SAs. Besides, the preparation process of ME inevitably introduces defects and adsorbates trapped between two materials, which lead to an increase in the non-saturation loss of the material [45].

## Experimental setup

**Fig. 8:**
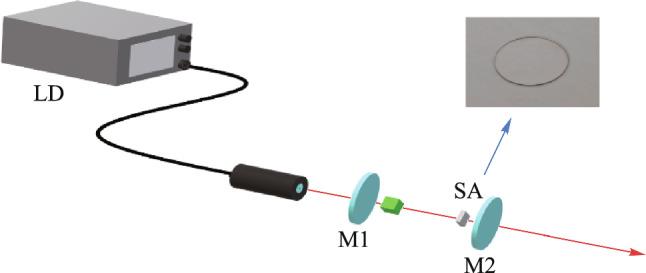
Experimental setup schematic diagram of the 2 μm all-solid-state Tm:YAP passive Q-switched laser

To verify the pulse modulation effects of SAs prepared by two different methods, a 2 μm all-solid-state Tm:YAP laser was set up, and Q-switched pulse outputs were achieved based on the two SAs, respectively. The experimental setup of the passive Q-switched laser based on BP/ReS_2_ heterojunction SA was shown in Fig. 8. It features a flat-concave cavity structure. A Tm:YAP crystal with a Tm^3+^ doping concentration of 3 at.%, as one of the most important crystals for 2 μm lasers due to its high gain, low threshold, wide absorption bandwidth, long fluorescence lifetime, high thermal stability, and excellent mechanical properties, was selected as the gain medium. It provides the basis for generating high-quality laser pulses. A 793 nm fiber-coupled laser diode (LD) was used as the pump source. The pumped light was focused through a coupling system with an imaging ratio of 1:1 to the center of the Tm:YAP crystal. Both surfaces of the crystal are coated with a 2 μm high-transmission (HT) film, and the incident side has an additional 793 nm HT film. The Tm:YAP crystal was wrapped in indium foil and mounted in a water-cooled copper block. The temperature is maintained at 20 °C through circulating water cooling. The planar mirror M1 is an incident mirror with HT film for 793 nm pump light and high reflectivity (HR) film for 2 μm lasers. The concave mirror M2 is an output mirror with a radius of curvature of 200 mm and a transmittance of 8% for 2 μm laser. Two types of BP/ReS_2_ heterojunctions were employed as SAs and positioned within the resonant cavity near the output mirror, respectively. A digital oscilloscope (Tektronix DPO 4102B-L) and a laser power meter (Thorlabs PM100D) were utilized to record the pulse waveform and measure the average output power, respectively.

## Experimental results

**Fig. 9:**
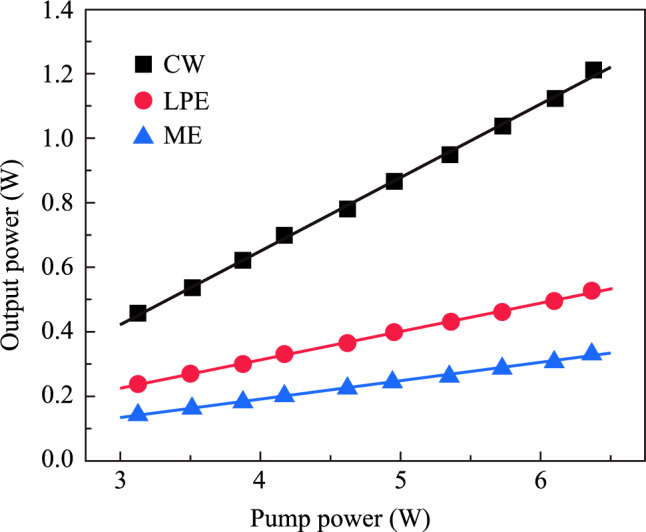
Average output power variation curve with respect to the pump power

By adjusting the resonant cavity and the pump power, the characteristics of the output laser were thoroughly investigated. When the pumping power was increased to 3.13 W, a stable Q-switched output was obtained. Figure 9 shows the pump power versus the average output power of the 2 μm all-solid-state Tm:YAP passively Q-switched laser based on BP/ReS_2_. The average output power exhibits a linear increase with the pump power. Without the SA, when the pump power reached 6.37 W, the maximum average output power of continuous wave (CW) laser is 1212 mW, corresponding to a slope efficiency of 23.1%. When the SA-LPE was placed in the cavity, the maximum average output power of 528 mW with a slope efficiency of 8.9% was obtained. Similarly, when the SA-ME was placed in the cavity, as shown in Fig. 9, the maximum average output power is 331.4 mW, with a slope efficiency of 5.8%. The main reason for the low output power of the laser with SA-ME is that the insertion loss of SA-ME is too high for its relatively large thickness.

**Fig. 10:**
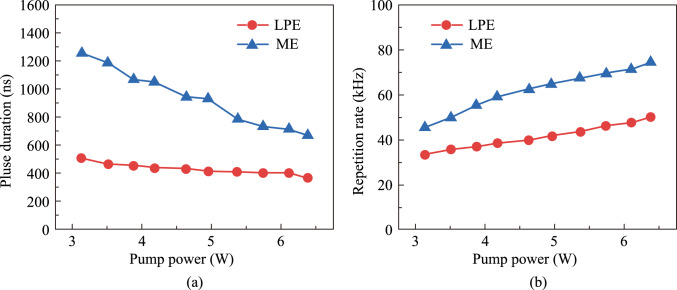
**a** Pulse width and **b** repetition frequency versus pump power

The pump power was gradually increased from 3.13 to 6.37 W. The pulse widths and repetition frequencies of SA-LPE and SA-ME were recorded and shown in Fig. 10a and b. The pulse widths all show a tendency to decrease with increasing pump power, with the range from 507 to 366 ns for laser with SA-LPE, and the range from 1256.5 to 671.25 ns for laser with SA-ME. As presented in Fig. 10b, the repetition frequency increases with the increase of pumping power, the range from 33.3 to 50 kHz and 45.5 to 74.5 kHz were obtained, respectively. This trend aligns with the typical characteristics of Q-switched pulse generation. Compared with the SA-ME, the SA-LPE employed in 2 μm all-solid-state lasers produces a narrow pulse width and small repetition frequency.

**Fig. 11:**
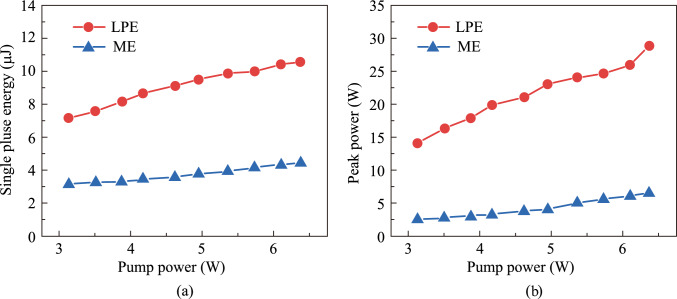
**a** Single-pulse energy and **b** peak power versus pump power

**Fig. 12:**
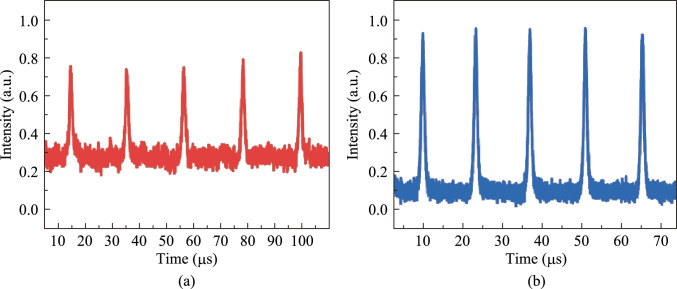
Multi-pulse sequences of laser with **a** SA-LPE and **b** SA-ME

According to the pulse width, repetition frequency, and average output power, the single-pulse energy and peak power of passively Q-switched pulses can be calculated. As shown in Fig. 11a and b, at the same pump power, the SA-LPE produces a larger single pulse energy and higher peak power compared to ME method. Figure 12a and b show the pulse sequences obtained from the SA-LPE and the SA-ME, respectively, at a pumping power of 6.37 W. Both pulse sequences exhibit good stability, which further demonstrates the excellent stability of the BP/ReS_2_ heterojunction in all-solid-state lasers. In contrast, the SA-ME exhibits slightly better pulse stability under the same conditions.

**Fig. 13:**
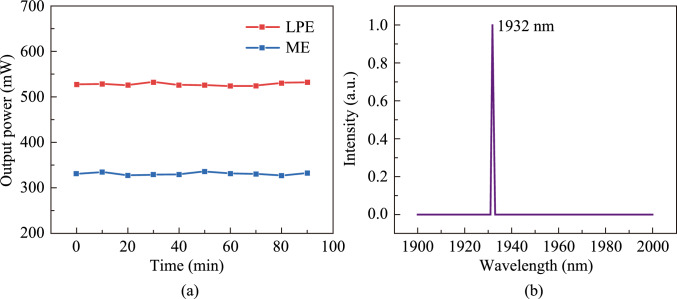
**a** Long-term stability of passive Q-switched lasers based on BP/ReS_2_ heterojunction SAs. **b** Output optical spectrum of Tm:YAP passively Q-switched laser based on BP/ReS_2_ heterojunction SA

The output powers based on different SAs were measured at 10-min intervals for 90 min as shown in Fig. 13a. For the Q-switched laser with SA-LPE, the maximum output powers of 533.4 mW and a minimum output power of 524.7 mW. For the Q-switched laser with SA-ME, the maximum output powers of 336.9 mW and a minimum output power of 327.7 mW. The output power fluctuation of both laser systems are less than 2%, indicating high stability. The long-term stability of the two laser systems were also investigated, stable Q-switched operation based on two types of BP/ReS_2_ heterojunction SAs were kept at least 3 h every day during five days.

The spectrum of the 2 μm Tm:YAP passively Q-switched laser based on BP/ReS_2_ heterojunction SA was measured, as shown in Fig. 13b, which exhibits the peak wavelength located at 1932 nm. The preparation method of SA has no significant effect on the spectrum of the laser.

The comparison with other 2D material-based saturable absorbers are presented in Table 1 [1, 39, 46–50]. As summarized in the table, this work based on BP/ReS_2_ heterojunctions has obtained a small pulse width and the highest peak power in the reported 2D SA passively Q-switched mid-infrared solid-state lasers. In particularly, compared with the pulse characteristics obtained by single BP SA or single ReS_2_ SA, the single-pulse energy and peak power obtained by BP/ReS_2_ heterostructures is obviously larger. Here, the tunable bandgap of BP effectively addresses the deficiency in the bandgap range of ReS_2_. BP/ReS_2_ heterostructures synergistically combine the advantages of BP (high carrier mobility and wide bandwidth) with those of ReS_2_ (excellent stability, switching ratio and photoresponsivity), demonstrating substantial research potential as SA for pulse modulation in the mid-infrared region.

**Table 1:** Comparison of laser characteristics with that of other 2D SAs

SA	Wavelength (nm)	Modulation depth (%)	Output power (mW)	Pulse width (ns)	Repetition rate (kHz)	Pulse energy (μJ)	Peak power (W)	Ref.
Graphdiyne	1985.8	11.74	770	785	199.6	3.86	5	[1]
BP	1988	–	151	1780	19.25	7.84	4.4	[39]
ReS_2_	2800	9.7	104	324	126	0.83	2.6	[46]
WSe_2_	2000	3.96	1510	427	82	12.8	30	[47]
PtTe_2_	2797	12	588	187	211	2.79	15	[48]
Graphene	1444	–	290	324	64	4.53	14	[49]
Bi_2_Te_3_	2000	7.5	1650	800	110	18.4	23	[50]
BP/ReS_2_	1932	11.5	528	366	50	10.56	28.85	This work

## Conclusions

In this study, BP/ReS_2_ heterojunction SAs were fabricated using LPE and ME method respectively, and their morphological structures and nonlinear absorption properties were systematically investigated. When applied in a Tm:YAP all-solid-state laser, both SA-LPE and SA-ME generates 2 μm passively Q-switched pulses. At a pump power of 6.37 W, the maximum average output power of 528 and 331.4 mW for SA-LPE and SA-ME were achieved, with the repetition frequency of 50 and 74.5 kHz, the pulse width of 366 and 671.25 ns, and the peak power of 28.85 and 6.63 W, respectively. These properties reveal the significant potential of BP/ReS_2_ heterojunctions for applications in pulsed lasers.

## Data Availability

The data that support the findings of this study are available from the corresponding author, upon reasonable request.
